# Non-pharmacological solutions to sleep and circadian rhythm disruption: voiced bedside experiences of hospice and end-of-life staff caregivers

**DOI:** 10.1186/s12904-018-0385-2

**Published:** 2018-12-22

**Authors:** Rana Sagha Zadeh, Elizabeth Capezuti, Paul Eshelman, Nicole Woody, Jennifer Tiffany, Ana C. Krieger

**Affiliations:** 1 Health Design Innovations Lab (affiliated with Cornell’s Institute for Healthy Futures), Design and Environmental Analysis, Cornell University, 2425 Martha Van Rensselaer Hall, Ithaca, NY 14853-4401 USA; 2 W.R. Hearst Foundation Chair in Gerontology, Hunter College of the City University of New York, New York, NY USA; 3 Healthcare Strategy & Operations Consultant, New York, NY USA; 4 Cornell University Cooperative Extension-NYC Programs, Outreach and Community Engagement, Bronfenbrenner Center for Translational Research, Ithaca, NY USA; 5 Center for Sleep Medicine, Weill Cornell Medical Center, New York, NY USA

**Keywords:** Advanced terminal illness, Palliative end-of-life care, Sleep disruption, Sleep/wake cycle, Symptom management, Caregiver experiences

## Abstract

**Background:**

Sleep disturbance is a significant issue, particularly for patients with advanced terminal illness. Currently, there are no practice-based recommended approaches for managing sleep and circadian disruptions in this population. To address this gap, a cross-sectional focus group study was performed engaging 32 staff members at four hospices/end-of-life programs in three demographically diverse counties in New York State.

**Methods:**

Participants responded to structured open-ended questions. Responses were transcribed and subjected to qualitative content analysis. The themes and recommendations for improved practice that emerged were tabulated using Atlas TI qualitative software.

**Results:**

This report details the experiences of hospice and end-of-life care staff in managing sleep and circadian disruptions affecting patients and analyzes their recommendations for improving care. Caregivers involved in the study described potential interventions that would improve sleep and reduce circadian disruptions. They particularly highlighted a need for improved evaluation and monitoring systems, as well as sleep education programs for both formal and informal caregivers.

**Conclusions:**

The voiced experiences of frontline hospice and end-of-life caregivers confirmed that disruption in sleep and circadian rhythms is a common issue for their patients and is not effectively addressed in current research and practice. The caregivers’ recommendations focused on management strategies and underscored the need for well-tested interventions to promote sleep in patients receiving end-of-life care. Additional research is needed to examine the effectiveness of systematic programs that can be easily integrated into the end-of-life care process to attenuate sleep disturbances.

## Background

Although often overlooked in care practice, disruptions in sleep and circadian rhythms are common issues experienced by a large percentage of individuals with advanced life-limiting illnesses [1–6]. The prevalence of sleep disturbance increases with age, comorbidities, chronic illness, disabilities, pain and arthritis, mental health disorders, institutionalization, and cognitive impairment [7]. Sleep disturbance results in depression, anxiety, fatigue, and a lower quality of life [8–10]. Specifically for patients, sleep fragmentation has been shown to increase symptoms including pain [11, 12], inflammation [13], delirium, cognitive decline [14], and risk of falls [15], ultimately contributing to a decrease in quality of life and an increase in healthcare costs [16, 17]. Patients’ sleep disturbances impact their caregivers by causing disrupted sleep for family members [18] and burnout for staff [19].

Systematic symptom-management practices related to sleep and circadian rhythms at the end of life are sparse, despite compelling data supporting behavioral approaches to improve sleep in other populations [20]. A 2006 study analyzing the available clinical practices for sleep disturbance in cancer patients found a gap between actual clinical practices and the existing recommendations in the literature [21]. That study certainly invites more emphasis on the translation-into-practice component of the research agenda about sleep disruption at the end of life (EOL), but it specifically focuses on the need for close examination of what exactly is happening in EOL care settings regarding sleep disruption. Caregivers’ perspectives on this topic are particularly useful: What are providers learning from their care-delivery experiences that can inform management about sleep disruption in EOL patients?

As a part of a larger project in New York State aimed to create educational programs for EOL caregivers about sleep and circadian management, the present study is intended to bring forward the voices of practitioners about their bedside experiences. In this study, we have collected the experiences of hospice and EOL caregivers in New York State related to sleep and circadian management. The results are intended to contribute to the long-term goal of developing holistic and comprehensive approaches to manage sleep and circadian disturbance for EOL patients by combining science with practice.

## Methods

### Study design

An ethnographic approach was chosen to collect bedside experiences of hospice and EOL staff caregivers and used a focus group methodology to facilitate interactive reflection.

The focus groups were held in 2016 and led by a professional moderator who asked the main questions following a written focus group guide and at least two investigators, who asked follow-up questions for clarification for each section. The focus group guide was developed based on an integrative review of 15 scientific databases [20]. This guide included six main sections with several subsections. The main questions were followed by additional probes about effective interventions to manage sleep and circadian disruption for individuals receiving EOL hospice care. Examples of probe topics include staff routines, qualities of physical environments, sleep hygiene strategies, and mind-body complementary health practices. In addition to demographic questions, we included questions about typical sleep problems, current treatments and practices, and solutions and ideal practices for the future. The final two categories of questions are the foci of this paper.

### Setting and participants

The study was conducted with hospice and EOL staff caregivers in four organizations. The participating facilities from Onondaga, Rockland, and Erie counties represented diverse populations in rural, suburban, and urban areas delivering care in all EOL settings, including hospitals, hospice residences, nursing homes, assisted-living communities, and private homes. Each year, these facilities provide palliative care services to a total of 5000 residents of Erie, Onondaga, Rockland, Oswego, and Madison Counties in New York State. These patients cover a wide age range (average age in the 70s), with terminal illness secondary to diverse medical conditions, including cancer, dementia, cardiac disease, neuromuscular conditions, pulmonary cirrhosis, and stroke. A total of 32 staff caregivers volunteered to participate, including physicians, nurses, social workers, chaplains, patient advocates, clinical leaders, and alternative-care therapists.

### Analysis

A qualitative content analysis was performed based on the methods of Lincoln and Guba [22–25]. Coding was done by two independently led teams from Cornell University and the City University of New York (a total of five people) using the Atlas TI software. Research team members had wide-ranging expertise, including palliative care nursing, education, public health, sleep medicine, health systems design, and health administration, which contributed to an interdisciplinary interpretation of the responses and enhanced the validity of the analysis. The four focus group studies were recorded and transcribed verbatim, resulting in a total of 237 pages of participant feedback.

Coding and content analyses were completed in three steps by two teams. At the end of each step, agreements were confirmed, and disagreements were compared and discussed until a consensus was achieved. The teams independently checked the coding to ensure that it was accurate, internally consistent, and mutually exclusive. The same analysis was then conducted on themes and subthemes that were created by clustering the codes. The final codes, themes, and subthemes were reviewed again by both teams, and the minor conflicts were resolved by consensus. During the analyses, members of each team compared results internally. Once teams achieved internal consensus, they shared their results with the other team. Saturation of themes was achieved, with all data fitting within the themes and subthemes and the new focus groups did not modify any codes or code relationships. Once the themes and subthemes were finalized, sample direct quotes from each main theme were selected to illustrate each topic. Although this is a qualitative study, the frequency of coding was quantitatively analyzed. Frequency does not denote importance of responses but only how often the voiced opinions were raised [26, 27].

Although the open-ended questions focused on current issues and interventions, participants also described a major need for a practical and systematic method to measure and evaluate sleep and circadian rhythms. These additions are reported in the results.

## Results

Each of the four focus groups contained 5 to 10 staff members per facility, with a total of 32 participants across all four facilities. The responses yielded 1003 statements (Table 1), categorized into 212 codes. These statements were divided into four distinct domains based on the primary stakeholder impacted by an identified intervention: (1) interventions involving interdisciplinary care teams, (2) interventions involving organizational leadership and management, (3) interventions involving environmental and technological resources, and (4) interventions involving national and state policymakers (Table 2).

**Table 1:** Frequency of each domain by focus group location

	Francis House	Hospice of Buffalo	Hospice of Central NY	United Hospice of Rockland	Total
Involving interdisciplinary care teams	206	106	204	132	648
Involving organizational leadership and management	13	42	66	57	178
Involving environmental and technological resources	36	27	19	47	129
Involving national and state policymakers	0	0	15	34	49

**Table 2:** Interventions to improve sleep/wake cycles and patients’ ability to fall asleep and stay asleep

Domain	Theme	Total
Interventions involving interdisciplinary teams		648
	Promote emotional and spiritual support (e.g., address family needs, provide comfort and peace, provide reassurance, and help with realistic expectations)	206
	Provide educational interventions to promote sleep	177
	Support individual circadian rhythms (e.g., adjust clinical processes and medications based on individual sleep assessments, determine causes of sleep problems, encourage a daytime routine, and avoid enforcing a traditional sleep/wake cycle)	168
	Promote physical comfort in patients	49
	Reduce negative stimulation and distraction	48
Interventions involving organizational leadership and management		178
	Provide patient-centered care (e.g., provide case managers and patient advocates to each patient in addition to dedicated staff members for particularly sensitive patients)	59
	Apply monitoring and staff feedback systems	43
	Provide disruptive and high-impact innovations that will lead to substantial improvements in multiple patients	38
	Accommodate alternative sleeping arrangements for patients	15
	Grant control over environment and amenities to staff, patients, and family	10
	Create internal policies to reduce disruptive noises	3
	Ensure that healthcare providers are trained to embrace each facility’s common philosophy	3
	Frequently measure, communicate, and document sleep quality and the impact of interventions	7
Interventions involving environmental and technological resources		128
	Optimize daytime environments (e.g., high-quality mattresses, daylight, soothing colors and scents, individualized sounds, and fresh air)	66
	Optimize night-time environments for sleep (e.g., keep rooms quiet and dark, eliminate odors, and provide white noise)	62
Interventions involving national and state policymakers		59
	Provide disruptive and high-impact innovations that will lead to substantial improvements in multiple patients	38
	Streamline national and federal reimbursement and care-related policies	11
	Dedicate more funding and resources to end-of-life and palliative care	10

### Interventions involving interdisciplinary care teams

At all four study sites, the most commonly discussed solutions for sleep issues in EOL care involved interdisciplinary care teams (Table 1). The most common themes within this domain included promotion of emotional and spiritual support, educational interventions, support for individuals’ circadian rhythms, promotion of physical comfort, and reduction of negative stimulation and distraction (Table 2).

#### Promotion of emotional and spiritual support

A considerable number of statements recommended that care teams take the following actions to create an emotional and spiritual environment supportive of restoration and sleep: reassure patients and family members about patient situations; discuss unrealistic expectations; align patient and family goals and expectations; advocate for family and staff presence; use strategies to help patients fall asleep (including relaxation techniques); provide dignity therapy, spiritual reassurance, and pastoral care; and prioritize patient and family needs over fixing sleep problems.

Several participants expressed that the emotional distress experienced by families and loved ones pose a barrier to restoration and sleep. One participant explained the patient experience: “Surrounded by your family home, you’re trying to come to terms with your mortality. Yeah. Mortality. The family’s holding on. ‘Are you okay? How do you feel? Will you eat?’ They can’t relax. They can’t sleep because they’ve always got this pressure.”

One interdisciplinary team member described the importance of addressing family needs for an emotionally comfortable and peaceful environment: “I think it’s 80 percent family and 20 percent resident … as far as family goes, a lot of them are very afraid. They don’t know what they’re seeing and what’s happening. If you can be part of that process too, that brings peace to the family, which makes the resident feel more comfortable.”

#### Educational interventions to promote sleep

Statements associated with this theme emphasized sleep education for staff, family members, and patients. The following directives emerged regarding professional staff:

- Staff should receive education including cultural competency related to sleep; up-to-date information on sleep, biology, and circadian rhythms; and case management and EOL patient care.
- Staff should be taught to optimize clinical decision-making by frequently evaluating the effectiveness of care and engaging in a holistic approach. Because a patient’s condition changes rapidly during EOL, such continuous evaluation will prevent under-prescription and over-prescription, which are detrimental to optimizing rest-wake rhythms.
- Staff should be taught to engage in patient-centered decision-making by providing some flexibility in decisions, adapting to patients’ existing needs and conditions, helping patients maintain a sense of control, adjusting to patients’ sensory capacities, and using patients’ native languages for communication.
- Staff should be able to decide in an educated manner between non-pharmacological and pharmacological interventions, avoiding unnecessary pharmacological interventions and taking patient preferences into consideration.

Other statements emphasized education of family members and patients about the importance of sleep, actions that can help manage sleep, and ways to modify the sleep environment to maximize comfort, as well as reassuring patients that it is safe for them to fall asleep.

Participants also explained that patient preferences should come first even if it means going against common rules, plans, or norms. One caregiver explained that the core of the intervention “is about the patient. Ask the patient [what her or his needs are and] … have the patient participate. Does the patient want bright lights? Does the patient want the television on? Or does it bother them? For some people, it might not bother them at all. It’s getting the patient involved in [her or his] environment. Some people have a preference.”

#### Support for individuals’ circadian rhythms

Respondents recommended that caregivers adjust clinical processes based on individualized sleep assessments. Caregivers should determine the underlying causes of sleep problems, avoid enforcing traditional sleep/wake cycles, and communicate effectively with family members about the process of supporting the patients’ needs and wishes. The respondents specifically recommended individualization of care practices in the following areas: adjusting patient medication, scheduling specific care procedures and visitations, and managing symptoms (especially pain). Furthermore, respondents recommended that caregivers take an individualized approach to planning meaningful daytime activities based on the patients’ habits and interests, encouraging patients to maintain a daytime routine that works for them, and continuously orienting patients to the time, weather, and day.

#### Promotion of physical comfort for patients

Focus group members recommended evaluation of patients’ physical comfort and identification of elements that interfere with sleep/wake cycles. Suggestions included offering physical therapy and pain-management techniques, massage, body pillows, warm showers, and warm beverages; addressing bowel issues and urinary comfort; and ensuring clean, dry bedsheets. One participant explained: “Sometimes, it’s just as simple as this person needs to void frequently throughout the night, so a catheter might help remove the issue for this case. Sometimes, it’s just as simple as that.”

#### Reduction of negative stimulation and distraction

Focus group members recommended that staff work to eliminate unnecessary distractions caused by family members. Examples include working with family caregivers to help them adjust to patient situations, providing family caregivers with positive activities and distractions so that patients can relax, screening phone calls to patients, and not placing phone ringers in patient rooms.

Staff should make efforts to minimize unnecessary care. For example, “[a] patient’s resting comfortably, but because an aide or a family member [is not informed about the importance of the comfort time], they’ll go in to, maybe to change them … turn them or something like that. They do it in such a manner that the patient’s come awake now. That’s a huge thing, not understanding to leave that, to respect patient comfort.”

### Interventions involving organizational leadership and management

Within the theme of patient-centered care, the focus groups recommended dedicating staff members to particularly sensitive patients and allocating patient advocates and a case manager to each patient. This organizational-level support will ensure the operationalization of the above recommendations, such as understanding and responding to individual needs.

Within the theme of monitoring and staff feedback, participants recommended the use of noninvasive technologies to monitor patients’ sleep/wake cycles and noise meters to alert staff of high noise levels. Suggestions for high-impact, noninvasive innovations that could lead to substantial improvements in patient care included creating simple, portable, non-pharmacological tools and technology to improve sleep and delirium and streamlining documentation and administrative processes. Within the theme of frequently measuring, communicating, and documenting sleep quality and the impact of interventions, the focus groups recommended documenting pre- and post-evaluation metrics, soliciting anecdotal feedback from patients and family members, and frequently discussing and informally assessing interventions. Other themes included accommodating alternative sleeping arrangements for patients; granting control over the environment and amenities to staff, patients, and family; creating internal policies to reduce disruptive noises; and ensuring that healthcare providers are trained to embrace a facility’s common philosophy about safeguarding sleep and circadian rhythms.

One participant explained the importance of a better system for measuring, communicating, and documenting sleep quality and the impact of interventions: “I think all the disciplines go in, and from visit to visit, so much of what we ask is the same, because of our medical record. If the nurses change medications, there’s a follow-up phone call that night, the next day, to monitor [whether it] is working. Each discipline is questioning what’s changed since [the] last visit. Sometimes a lot of it is anecdotal.”

### Interventions involving environmental and technological resources

Within the theme of optimizing daytime environments, the focus groups recommended that environments include comfortable high-quality mattresses, soothing color palettes and scents, flexibility in room design, visual elements, operable windows for fresh air, individual climate control, and private bathrooms. In addition, respondents suggested that facilities allow pets, remove mirrors in patient bedrooms, and allow personal and religious items.

One focus group member explained that it is very important, if possible, “to control the ambient temperature [and] have fresh air.” Another member added that “the equipment does also help. I mean a good bed with the right mattress [and] a commode.”

The focus groups suggested that facilities optimize nighttime environments for sleep by offering white noise, controlling unwanted noise (e.g., ensure that rooms are designed to be quiet and dark, soundproof walls/floors, ensure that phone ringers are off or screen phone calls, place TVs close to patients’ heads, and provide call buttons), controlling unwanted light (e.g., provide dimmable lights and blackout shades), eliminating odors, bringing fresh air into patient rooms, providing private spaces outside of patient rooms for family members, and granting care teams greater control over patient room equipment. One participant explained: “Any noise, conversation, and smell can be potentially disturbing. As soon as somebody smells bacon, they’re awake, so you want to be careful when you put it on. That’s a little thing, but it’s true.”

Within the theme of positively disruptive, high-impact innovation that will lead to substantial improvements in multiple patients, the focus groups recommended creating simple, portable, non-pharmacological tools and technology to improve sleep and delirium and streamlining documentation and administrative processes. One participant discussed the potential use of iPhone technology to customize sleep management techniques for each patient and to monitor disruption: “A sleep ‘*Siri’* … get initial input so they can personalize [it], but also be able to continue to record and go back to it.”

### Interventions involving national and state policymakers

Participants explained that a thorough improvement in patient outcomes regarding sleep symptom management will be possible only by improving policies related to restricting pain medication and reimbursement, as well as referral policies surrounding EOL and palliative care.

One participant commented: “A hospital we know does not give pain medication. The pain management consultant does not give pain medication. They don’t wanna be audited. They don’t want to do anything that will raise a red flag, so that’s really a policy issue.” Another person commented: “Primary care needs to take more of an active lead in palliative care management because there’s not a payer source for palliative care. There’s a big gap between primary care and hospice care.” This current U.S. payment policy gap in which palliative care is not reimbursed prior to hospice eligibility (requiring documentation of a 6-month prognosis) directly impacts how symptoms such as sleep and pain are evaluated or treated in the primary care setting.

## Discussion

Collectively, the experienced EOL caregivers shared numerous strategies for managing disturbance in sleep and circadian rhythms, many of which can be easy to apply if coordinated with all stakeholders. The results of this study revealed four main unmet needs that require further attention to improve sleep disturbance in patients with advanced terminal illnesses in hospice settings. All share an educational dimension and call for the development of ongoing programs that combine experience-based and evidence-based knowledge regarding non-pharmacological interventions. These unmet needs fall into four domains: (1) better integration of person-centered, interdisciplinary care; (2) engagement of organizational leadership and management teams; (3) improvement of environmental and technological support; and (4) involvement of regional and national policymakers to address this issue on a larger scale and avoid potential liability at an institutional level.

Within the first domain, there is a need for interdisciplinary educational strategies and teams with complementary expertise and training in managing sleep disturbance in order to guarantee that staff caregivers feel comfortable having conversations with patients and families [28]. Participants described commonly used interventions (physical comfort, emotional and spiritual support) that can promote sleep but were concerned about how to communicate with families about patient-centered decisions regarding comfort and scheduling to maintain sleep-wake integrity. They felt that family caregivers, in their efforts to provide the best care, act *for* the patient instead of *with* the patient. There may also be a lack of concordance between the perceptions of family caregivers and patients regarding sleep and fatigue [29]. The literature identifies three dimensions of family functioning in the EOL context: family cohesion, expressiveness (ability to discuss feelings), and conflict resolution [30]. Dysfunction in any of these dimensions may result in family caregivers exhibiting behaviors such as protectiveness in an effort to reduce distress and sustain hope [31, 32]. Similarly, family dysfunction may compel the patient to be protective of their families [32]. For instance, patients might not want to ask family members to leave the room when they are sleepy [32].

The importance of educational programs on sleep management was highlighted. Participants felt that if they were more knowledgeable about sleep and circadian rhythms, they would be better able to educate families on caregiving approaches. Educating families about patients’ individual circadian rhythms and how these may have changed due to advanced illness would hopefully reduce well-meaning disruptions from family members. Because not all family caregivers would be receptive to changing their approach, education should include how to facilitate patient-family discussion, how to assess family functioning, and when to refer patients and families for additional psychological assistance [30]. For some families, tailored coping and communication support intervention [33] may be indicated.

The second domain pertains to team management and organizational leadership interventions that provide the necessary expertise for care coordination and personalization and the resources and tools to ensure that patients and caregivers get support and guidance while integrating sleep into the care model. A need for more systematic evaluation of sleep was highlighted. One evaluation of 150 patients with breast and lung cancer (various stages) showed that although 44% had frequent sleep problems, they were never asked about their sleep [34].

In the third domain, participants described the optimization of the sensory environment for both day and night and the related technological resources. Among the recommended interventions involving environmental and technological resources, the emergent theme was “control”: How can educational materials, procedures, and policies for EOL care facilities enable the greatest possible degree of control? Such control, in this case related to sleep patterns, is necessary to accommodate the highly individualized, continuously changing needs of each patient; to help loved ones find the balance of respite and connection to grieve and be present for and supportive of the patient; and to enable care staff to be most effective in minimizing disturbances to sleep patterns or developing daily wakefulness strategies. One key environmental support is the use of single-occupancy rooms in institutional settings [35–37]. Research is needed to better understand how much, and what types of, control to provide to patients and how to use facility design to reconcile the varying and potentially conflicting needs of patients, families, and staff in order to best manage patient symptoms.

The fourth domain called for engagement from health policymakers to ensure that educational strategies, protocols, and support are available to caregivers, facilities, and staff to empower them to make appropriate decisions regarding allocation of resources and programs to protect sleep in EOL patients. Palliative and hospice services are successful in reducing barriers to pain relief, such as the fear of addiction and shortening of life [38]. Thus, regulations that target the current debates on opioid misuse should not decrease access to appropriately prescribed pain management in the terminally ill as found in other countries [39]. Prevention of disruption of sleep and circadian rhythms is closely tied to effective management of other symptoms, such as pain.

## Conclusion

The results from this study can inform care protocols, policies, procedures, and the research agenda. Many of the interventions described mirror the principles of sleep hygiene [20], which are behavioral and environmental practices aimed to promote healthy sleep habits (e.g., eliminate noise from the sleeping environment, maintain a regular sleep schedule, stress management, avoidance of caffeine, nicotine, alcohol, and daytime napping). Participants, however, acknowledged the difficulty in enacting these interventions when family and staff caregivers take control of the patients’ environment and activities. The participants emphasized a need for easy-to-implement, high-impact, and customizable ways to optimize patient care to minimize sleep disturbances.

Further research on improving education, developing guidelines, and sharing data will allow improved strategic planning and implementation of policies and procedures focused on a better sleep experience for patients with terminal illnesses in hospice care.

## Abbreviations

EOL: End of life
